# *Ligustrum robustum* Intake, Weight Loss, and Gut Microbiota: An Intervention Trial

**DOI:** 10.1155/2019/4643074

**Published:** 2019-04-14

**Authors:** Tao Zhou, Jiayi Chen, Yuhang Chen, Jiayi Xu, Sijing Liu, Tianli Zheng, Dianjianyi Sun, Lu Qi, Jingyuan Xiong, Xiaofang Pei

**Affiliations:** ^1^Department of Public Health Laboratory Sciences, West China School of Public Health, Sichuan University, Chengdu 610041, Sichuan Province, China; ^2^Department of Epidemiology, School of Public Health and Tropical Medicine, Tulane University, New Orleans, LA, USA; ^3^No. 4 West China Teaching Hospital, West China School of Public Health, Sichuan University, Chengdu 610041, Sichuan Province, China; ^4^Public Health and Preventative Medicine Laboratory Training Center, West China School of Public Health, Sichuan University, Chengdu 610041, Sichuan Province, China; ^5^Department of Nutrition, Harvard T.H. Chan School of Public Health, Boston, MA, USA; ^6^Channing Division of Network Medicine, Department of Medicine, Brigham and Women's Hospital and Harvard Medical School, Boston, MA, USA

## Abstract

*Ligustrum robustum* (*LR*) shows antiobesity effects in animal studies. However, little is known about the effect on human. The present study aimed to investigate the effect of* LR* intake on weight change in obese women and the role of gut microbiota. Thirty overweight and obese female participants (BMI ≥24 kg/m^2^) were recruited in the current study. The participants drank* LR* 10g/d for 12 wks. Their body composition and related biomarkers were assessed. Alterations of the gut microbiota were analyzed using 16S rRNA sequencing. The primary outcome was the change in body weight.* LR* intake resulted in 2.5% weight loss over 12 wks (P<0.01). Change in body fat at 12 wk was -1.77 ± 1.19 kg (P<0.01). In addition, decreased Firmicutes-to-Bacteroidetes ratio (P=0.03), increased richness (the ACE estimator, P<0.01; the Chao1 estimator, P<0.01), and altered representative taxa of the gut microbiota were observed.* Bacteroidaceae, Bacteroides*,* Bacilli*, and* Lactobacillales *were higher while* Ruminococcaceae, Enterobacteriaceae, Enterobacteriales, Lachnospiraceae, Clostridia, *and* Clostridiales *were lower at 12 wk. Moreover,* LR* intervention decreased fasting glucose (P<0.01), serum leptin (P<0.01), and IL8 (P=0.02) and increased HOMA-*β* (P<0.01).* LR* intervention moderately decreased the body weight in overweight and obese women and such effect might be due to modulation of gut microbiota.

## 1. Introduction

Obesity has become an epidemic worldwide [1, 2]. Gut microbiota has recently emerged as a novel, metabolically active organ, tightly linked to obesity [3, 4], through various mechanisms such as the production of bioactive compounds influencing fat storage and metabolism [5]. Thus, dietary modulation of gut bacteria is considered to be a promising method for preventing and treating obesity.

*Ligustrum robustum* (*LR*), a traditional Chinese beverage plant, contains abundant bioactive compounds with health benefits, such as flavonoids, total phenolic acid, polysaccharide, and triterpenoid [6, 7]. Animal studies have shown the antiobesity, antioxidative, anti-inflammatory, antitumor, and hepatoprotective effects of* LR *[8–10]. Moreover, the* LR*-induced changes in the composition of gut microbiota were also observed accompanied with weight loss in an animal study [11].

Although accumulating data highlight the potential effects of* LR* on weight loss, the direct evidence from human studies is lacking; and little is known about whether* LR* affects gut microbiota in humans. Because women have a higher body fat percentage than men and are more likely to suffer from obesity and are more likely to want to lose weight [12], in the current 12 wk intervention trial, we aimed to evaluate the effect of* LR* on body composition and gut microbiota among overweight and obese women.

## 2. Materials and Methods

### 2.1. Components Analysis of LR

To preparation of* LR* extract for component analysis, 50 g* LR* was ground to a fine powder and suspended in 1500 mL distilled water and incubated with shaking at 80°C for 3 h. Then the extract was filtered and further evaporated on a rotary evaporator to a concentration to 1 g/mL and stored at −20°C until further use [10]. The analytical solution was prepared by filtering 220 *μ*L supernatant using a 0.22 *μ*m filter.

An ACQUITY UHPLC system (Waters Corporation, Milford, USA) coupled with an AB SCIEX Triple TOF 5600 System (AB SCIEX, Framingham, MA) was used to analyze the metabolic profiling in both ESI positive and ESI negative ion modes. An ACQUITY UPLC BEH C18 column (1.7 *μ*m, 2.1 × 100 mm) was used in both positive and negative modes. The analysis was performed in binary gradient mode, with water (containing 0.1% formic acid, v/v) as mobile phases A and acetonitrile (containing 0.1% formic acid, v/v) as mobile phases B. The separation was achieved using the following gradient: 0 min, 5% B; 2 min, 20% B; 4 min, 25% B; 9 min, 60% B; 14 min, 100% B; 18 min, 100% B; 18.1 min, 5% B; and 19.5 min, 5% B, with a flow rate of 0.4 mL/min and column temperature of 45°C. All the samples were kept at 4°C during the analysis. Full scan mode (m/z ranges from 70 to 1000) combined with IDA mode was used in data acquisition. The ion source temperature was 550°C for positive and negative ion mode. The ion spray voltage was 5500 V for positive ion mode and 4500 V for negative ion mode. For IDA analysis, the range of m/z was from 25 to 1000 and the collision energy was 30 eV. The injection volume was 2 *μ*L. The measurement was performed by Shanghai Lu-Ming Biotech Co., Ltd., Shanghai, China. Raw data was analyzed by Progenesis QI software (Nonlinear Dynamics, Newcastle, UK).

### 2.2. Subjects

Community female volunteers aged 30 to 60 years and whose body mass index (BMI) over 24 kg/m^2^ were invited to join the study [13]. Subjects who were using medications that affect body weight, having a history of chronic illness, taking antibiotic within 3 months, changing their diet recently, or showing insufficient motivation to complete the study, were excluded. Thirty-six volunteers attended the first interview. One volunteer was excluded for basic disease, four for BMI <24 kg/m^2^ and one volunteer dropped out because of work. Demographic characteristics of participants including age and medical history were obtained by questionnaire.

The Medical Ethics Committee of Sichuan University approved the study (Approval No. K2016025). Written informed consent was obtained from all participants. This trial was registered at http://www.chictr.org.cn (ChiCTR-OOh-17011832).

### 2.3. LR Intervention

Processed leaves of* LR* were obtained from China's largest* LR* provider, Green Hills and Blue Waters Co., Ltd. (Junlian, Sichuan, China). One tea bag was packed with 5g grounded* LR* leaves. No additional chemical was introduced during preparation of the tea powder. One tea bag was brewed with 420 mL boiled water for at least 10 minutes for one cup of tea. Additional hot water was permitted for the same tea bag when needed.

Participants were instructed to drink* LR* twice per day after meals for 12 wks. Participants were told to consume their habitual diets throughout the study. Other tea and antibiotic were prohibited in the study. At the end of the intervention, the number of tea bags left was counted to evaluate the compliance.

### 2.4. Anthropometric Measures and Biochemical Analysis

Body composition measurement was introduced by multifrequency BIA with eight tactile electrodes (InBody 720; Biospace, Seoul, Korea). Body weight, abdomen circumference, hip circumference, and body composition (i.e., body fat, visceral fat area, body protein, and body muscle) were measured in the morning with light clothing before breakfast with an empty bladder at baseline, 4 wk, and 12 wk. Height was measured without shoes. Reduced weight (%) was defined as [(initial body weight - body weight at follow-up)/initial body weight] × 100 [14]. Triglyceride (TG), total cholesterol (TC), low-density lipoprotein (LDL), high-density lipoprotein (HDL), insulin, C-Peptide, glucose, and biomarkers for safety assessment were assessed by an automatic biochemical analyzer in No. 4 West China Teaching Hospital at baseline and 12 wk. We further calculated homeostasis model assessment of insulin resistance (HOMA-IR) by fasting insulin (mU/L) ×fasting glucose (mmol/L)/22.5 and HOMA of *β*-cell function (HOMA-*β*) by 20× fasting insulin (mU/L)/[fasting glucose (mmol/L)-3.5] ×100%. Fasting blood samples were stood for 30 minutes to clot before centrifugation for 10 minutes at 1000×g. Then the serum samples were frozen at −80°C until analysis. Moreover, we measured leptin, interleukin-6 (IL6) and interleukin-8 (IL8) using the LUMINEX technology (Millipore, Billerica, MA, USA).

### 2.5. Dietary Assessment and Physical Activity Monitor

To evaluate the dietary adherence, we collected food frequency questionnaires (FFQs) which measured the frequency and quantity of major food and beverages intake from all participants at baseline and 12 wk [15]. Physical activity was evaluated at baseline and 12 wk by International Physical Activity Questionnaire (IPAQ) [16]. The frequency and duration of various types of physical activity were obtained from participants. The metabolic equivalent (MET) value was defined as the ratio of the work metabolic rate to a standard resting metabolic rate (RMR). One MET is defined as 1.0 kcal/kg/hour [17]. We multiplied the corresponding MET value by the number of hours per week involved in the activity to get an average energy expenditure for each activity.

### 2.6. 16S rRNA Sequencing

The microbial communities of 29 participants were analyzed by high-throughput sequencing. Fecal samples collected at baseline and 12 wk were immediately stored at -80°C for gut microbiota analysis. DNA of each fecal sample was extracted by using the QIAamp Fast DNA Stool Mini Kit (Qiagen, Hilden, Germany). The DNA concentration was determined by Nanodrop spectrophotometer (NanoDrop 2000, Wilmington, USA). V4 region of 16S rRNA was amplified using primer 515F (5′-GTGCCAGCMGCCGCGGTAA-3′) and 806R (5′-GGACTACHVGGGTWTCTAAT-3′) with the barcode. Sequencing was performed on an Illumina Miseq platform (Novogene, Beijing, China) based on a standard protocol from the manufacturer. Raw sequences were processed by using the QIIME software package. Sequences with ≥97% similarity were assigned to the same OTUs. The representative sequence for each OTU was screened for further annotation.

### 2.7. Adverse Event Monitoring

Adverse events associated with* LR* intake were self-reported by the participants. Additionally, biomarkers such as aspartate aminotransferase (AST) and alanine aminotransferase (ALT) in serum were measured to evaluate liver function.

### 2.8. Statistics

All statistical comparisons are 2-sided. The level of significance for all tests was set to P<0.05. Baseline data are reported as means ± standard deviation or median and interquartile range when appropriate. The concentration of biomarkers only available at baseline and 12 wk was analyzed using paired T-test for normal distribution variables and Wilcoxon signed-rank test for nonparametric tests. Above statistical analyses were performed with SAS version 9.4 (SAS Institute Inc., Cary, NC). Linear discriminant analysis coupled with effect size (LEfSe) was used to identify the taxa differentially represented between baseline and 12 wk. Alpha diversity, Principal Coordinate Analysis (PCoA) and clustering in our samples were calculated and displayed with R software (Version 3.3.3). The study was powered to detect a 0.5 kg weight loss as an effect of LR intake over the intervention. Power calculations were performed with G*∗*Power 3.1.

## 3. Results

### 3.1. Characterization of LR Composition

A total of 7477 components were identified in* LR*. The most abundant top 25 components of both positive ion mode and negative ion mode were shown in Table 1.

### 3.2. Characteristics of Study Participants

Characteristics of participants are shown in Table 2. At baseline, the average age of the participants was 47.9±5.4 years, the mean BMI was 29.85±3.70 kg/m^2^, and the mean percentage of body fat was (41.33±5.27)%. Participants had higher physical activities at baseline than at 4 wk and 12 wk (P=0.01). No significant change in dietary energy intake was found across the 12-wk study (P=0.62).

### 3.3. Effects of LR on Body Composition

Participants showed significant weight loss in response to* LR* intervention (P < 0.01). The average reduced weight (%) was 0.6% at 4 wk and 50% of the participants achieved a weight loss of 2.5% at 12 wk. In addition, the changes in fat from baseline to 12 wk were -1.77 ± 1.19 kg (P <0.01). Corresponding change in the visceral fat area was -5.25 cm^2^ (P <0.01). Change in abdomen circumference was significant at 12 wk (P=0.01). No significant changes were observed in hip circumference, body protein, and muscle (Table 2).

### 3.4. Metabolic Biomarkers

The change of biochemical markers between baseline and 12 wk was analyzed (Table 3). There were significant decreases in serum leptin, fasting glucose, and IL8 at 12 wk compared with baseline (all P<0.05). We also observed a significant increase in HDL. No changes in TC, TG, LDL, insulin, and C-Peptide were observed. In addition, HOMA-*β* increased at 12 wk (P<0.05). Moreover, except decreases in ALT and AST, other parameters did not differ between the baseline and 12 wk (Table 3).

### 3.5. Effects of LR on Gut Microbiota

**Figure 1:**
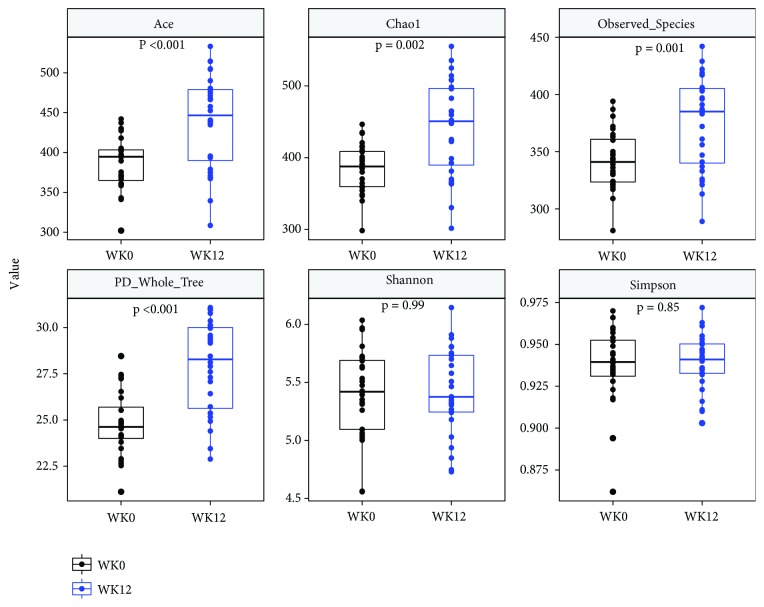
Alpha diversity measured by ACE, Chao 1 index, observed species, PD whole tree, and Shannon and Simpson index. WK0, baseline; WK12, 12 wk.

The gut microbiota was dominated by* Firmicutes*,* Bacteroidetes,* and* Proteobacteria* at the phylum level across the study. A greater community richness was shown at 12 wk compared with baseline (the ACE estimator, P<0.01; the Chao1 estimator, P<0.01). We also detected a significant difference at the observed species level (P<0.01) and increased phylogenetic diversity (PD_whole_tree, P<0.01), while there is no significant change in Shannon (P=0.99) and Simpson (P=0.85) indices (Figure 1).

**Figure 2:**
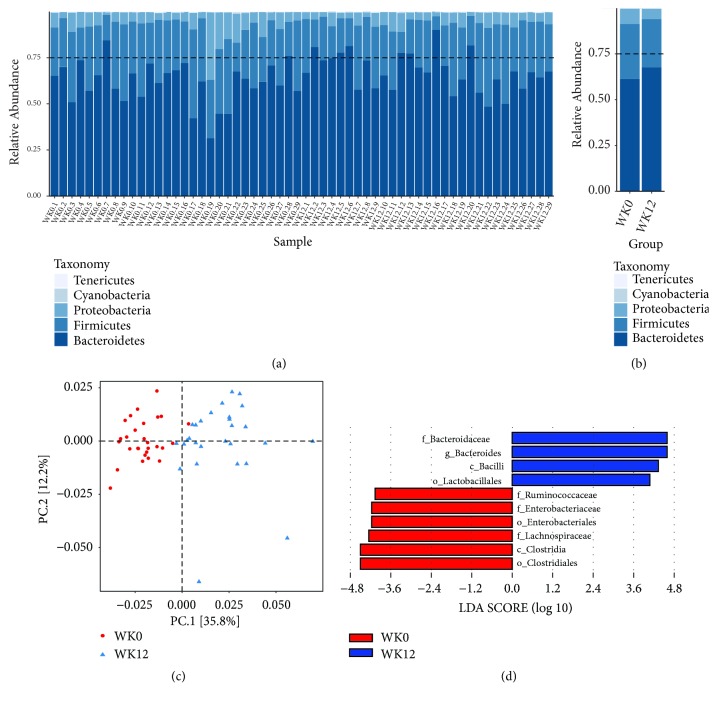
Modifications in gut microbiota after* Ligustrum robustum* (baseline and 12 wk). (a)-(b) 16S rRNA sequencing analysis and taxonomy classification of the gut microbiota at the phylum levels. (c) Principal coordinate analysis (PCoA) of the microbial community structure before and after* Ligustrum robustum* drinking (N=28). The analysis was based on the abundances of all class of bacteria acquired by high-throughput sequencing by unweighted UniFrac analysis. The bacterial community of baseline was represented in red circle and 12 wk in blue triangle. (d) Linear discriminant analysis (LDA) effect size (LEfSe) identified taxa most characteristic (increased and decreased abundance) at 12 wk. c_, class; o_, order; f_, family; g_, genus; WK0, baseline; WK12, 12 wk.

We found a significantly decreased* Firmicutes*-to-*Bacteroidetes* ratio before and after* LR* intervention (0.53 versus 0.42, P=0.03) (Figures 2(a) and 2(b)). We further analyzed the beta diversity of gut microbiota. PCoA showed distinct bacterial community structures between baseline and 12 wk (Figure 2(c)). We also identified microbial taxa which accounted for the differences between baseline and 12 wk. In comparison with those at baseline,* Bacteroidaceae, Bacteroides*,* Bacilli*, and* Lactobacillales *were higher while* Ruminococcaceae, Enterobacteriaceae, Enterobacteriales, Lachnospiraceae, Clostridia, *and* Clostridiales *were lower at 12 wk (Figure 2(d)).

We further examined whether the gut microbiota differs between those achieving a 2.5% weight loss and those not; however, no significant difference was found on the composition of gut microbiota.

### 3.6. Safety Assessment

No participants withdrew from the study for discomfort or adverse effects in response to the invention. According to the follow-up, though 2 participants reported mild abdominal pain at the first 2 days, the discomfort was soon relieved. After the study, 6 participants (including 1 participant who had reported mild abdominal discomfort) reported a beneficial effect of* LR* on promoting defecation. In addition, AST and ALT decreased after the intervention. No difference was found for other measurement of safety assessment.

## 4. Discussion

In the current study, we found that* LR* intervention for 12-wk showed favorable impact on weight, body fat, visceral fat area, HDL, leptin, and abdomen circumference among overweight and obese female participants. In addition, we found that* LR* intervention decreased the Firmicutes-to-Bacteroidetes ratio, increased richness, and altered representative taxa of the gut microbiota.

Our observation in overweight and obesity middle-aged females is in line with our previous animal study, in which we found that* LR* extractive intake decreased weight and changed gut bacteria in rat [11]. A recent mice study also showed the antiobesity effects of phenylpropanoid glycosides extracted from* LR *[9].

Gut microbiota has been demonstrated to be a cause of obesity [3]. Efficacy of energy harvest from food can be affected by the composition of gut microbiota [18]. Modifying the intestinal microbial community by diet has become a new approach to prevent and treat obesity [4, 19, 20]. Obesity has been associated with a low bacterial richness [21, 22]. Dietary and several natural beverage plants have shown modification on the composition of gut microbiota [18, 23, 24]. In the present study, we found that richness of the gut microbiota was higher at 12 wk than baseline, and this change was similar to the effect caused by energy-restricted diet intervention and Roux-en-Y gastric bypass surgery [18, 25]. Though mechanisms remain unknown, our data suggest that bioactive compounds such as plant polysaccharides contained in* LR *[7] may partly account for the effects, and such postulation is supported by the study conducted by De Filippo et al., in which diet nutrients were associated with bacterial richness and diversity [26].

We also found that drinking* LR* for 12 wks resulted in a significant decrease in the ratio of Firmicutes to Bacteroidetes (F/B ratio). A study showed that, in obese individuals, the F/B ratio was higher than that of lean individuals. The increase of the abundance of* Bacteroides* was associated with the percentage of weight loss caused by fat restricted or carbohydrate restricted diet [20]. Bariatric surgery was also found to diminish the F/B ratio during weight loss [27]. Thus, the decreased body weight observed in our study might be related to the regulation of F/B ratio [20, 28]. In addition, bacterial communities clustered according to prior and after* LR* intervention, highlighting the fact that the enrichments and diversity of gut microbiota were affected by* LR *[29]. Furthermore, human twins study revealed that obesity was associated with the altered representation of bacterial genes [22]. We showed a significantly different abundance of specific taxa between baseline and 12 wk. We noted an increase in* Lactobacillales* group (which was in line with our previous rat study) and a decrease in* Clostridiales* and* Lachnospiraceae* groups with the same trend as other weight loss related studies described [30]. A previous study has shown that excessive fat accumulation was associated with a low-grade chronic inflammatory [31]. The endotoxin activity of LPS from members of the families* Enterobacteriaceae* is 1000-fold higher than that from* Bacteroidaceae *[3]. As an endotoxin, LPS is the major component of Gram-negative bacteria that causes inflammation related to obesity. We observed an increase in* Bacteroidaceae* and reduction of* Enterobacteriaceae*. Therefore, the anti-inflammatory effect of* LR*, which was further confirmed by the downregulation of IL 8 [32], might also account for weight loss induced by* LR* intervention.

Another potential mechanism of the weight loss effect induced by* LR* may be through the improvement in metabolic biomarkers. A previous in vitro study demonstrated that* LR* extract could downregulate the activities of *α*-amylase and *α*-glucosidase in Caco-2 cell monolayer [33]. Α-amylase and *α*-glucosidase are associated with starch and glycogen digestion and glucose absorption, respectively [34, 35]. Evidence suggests that weight loss is associated with improvement of insulin resistance, *β* cell function, and a reduced risk of developing type 2 diabetes [36–38]. The increase in HOMA-*β*, together with the decreases in fasting glucose after the trial, suggest a potential role of* LR* in control of postprandial hyperglycemia. As the product of the adipose-specific* ob* gene, leptin is secreted by adipose tissue and plays a role in regulating food intake and energy expenditure. Compared with lean people, the concentration of leptin in the peripheral circulation is approximately fourfold higher in obese people and this may lead to leptin resistance [39]. We observed a decrease of leptin along with weight loss, which was in accordance with the study performed by May Faraj [40]. With a reduction in leptin level in our study,* LR* intervention presented a potential role in downregulating energy store [41]. However, our result was different with a previous mice study in which the antiobesity effect caused by* LR* was associated with increased leptin [9]. This discrepancy may be due to the different study subjects.

To our knowledge, this is the first intervention trial on the effect of* LR* on the changes in weight, body composition measures, and other obesity-related biomarkers among overweight and obese middle-aged Chinese women. A major strength is that we analyzed the role of* LR* intervention on the modulation of gut microbiota. However, several limitations should be considered. First, because the flavor of* LR* is quite bitter, it was difficult to find an ideal placebo to conduct a blinded controlled study. Lack of participants as a control group and the small sample size might lead to uncertainty in our estimates. Second, we only performed analysis in women; further studies in men are needed. Third, data of contraception method which might influence the related measurement was not obtained in the current study. Fourth, we measured body composition by BIA, which is less reliable than dual-energy X-ray absorptiometry. Also, weight loss effects of* LR* in different doses warrant further studies.

Taken together, this study suggests that daily drinking of* LR* 10g per day for 12 weeks may improve body weight. This favorable outcome is associated with the alterations of gut microbiota and metabolic status. Our results support consumption of the* LR*, a traditional beverage, in the treatment of obesity.

## Figures and Tables

**Table 1:** Characterization of chemical compounds of *Ligustrum robustum*.

ID	m/z	Rt(min)	Metabolites	Formula	Me (ppm)	Rc
*Positive mode*						
1	256	11.79	3-Methylcyclopentadecanone	C16H30O	-2.91	392814266.4
2	280	11.56	2-(5,8-Tetradecadienyl)cyclobutanone	C18H30O	-0.69	189789298.9
3	152	1.20	L-Malic acid	C4H6O5	3.48	88867512.6
4	338	12.59	13Z-Docosenamide	C22H43NO	-3.19	88330756.9
5	338	12.05	13E-Docosenamide	C22H43NO	-4.16	83549663.1
6	136	0.69	4-Hydroxy-L-threonine	C4H9NO4	2.44	69818264.2
7	129	13.31	cis-3-Chloroallyl aldehyde	C3H3ClO	-3.71	55202432.5
8	579	6.84	RHOIFOLIN	C27H30O14	-1.14	48591563.5
9	247	11.85	5,7-Diethyl-9-methyl-3E,5E,7E,9E-tridecatetraene	C18H30	-0.81	38479884.8
10	310	12.19	3,4-Epoxy-6Z,9Z-eicosadiene	C20H36O	2.35	26476464.8
11	163	5.23	4-Hydroxycoumarin	C9H6O3	-4.18	23461225.0
12	303	6.49	Delphinidin	C15H10O7	-4.26	21141278.3
13	455	7.24	Ampelopsin D	C28H22O6	0.65	20516556.0
14	312	10.97	8E-Heneicosene	C21H42	-1.90	18469775.4
15	284	1.19	Guanosine	C10H13N5O5	-3.09	17980999.0
16	309	5.74	p-Coumaroyl-D-glucose	C15H18O8	-2.65	16642683.2
17	611	6.48	Rutin	C27H30O16	-0.83	16302462.1
18	151	5.71	4-Hydroxyphthalide	C8H6O3	-4.75	16197253.7
19	147	6.50	3(S)-hydroxy-13-cis-docosenoyl-CoA	C4H4ClN3	-1.02	15818105.7
20	243	5.86	3-Furanmethanol glucoside	C11H16O7	-3.69	15286833.3
21	309	9.04	6-O-p-Coumaroyl-D-glucose	C15H18O8	-2.11	15264518.9
22	163	4.25	UMBELLIFERONE	C9H6O3	-0.74	15248352.7
23	455	7.90	Benzyl gentiobioside	C19H28O11	3.86	11520143.1
24	284	11.68	Octadecanamide	C18H37NO	-2.35	10503591.6
25	309	6.91	3-Hydroxy-4,6-heptadiyne-1-yl 1-glucoside	C13H18O7	3.03	10093831.0
*Negative mode*						
1	737	7.16	N-Deisopropyl-fluvastatin	C21H20FNO4	-3.49	161866859.3
2	785	6.28	Echinacoside	C35H46O20	-0.09	93138299.4
3	625	8.07	PI(22:6(4Z,7Z,10Z,13Z,16Z,19Z)/0:0)	C31H49O12P	3.38	64107509.6
4	769	6.87	Nilvadipine	C19H19N3O6	2.80	63734793.5
5	753	7.09	Kinetin-7-N-glucoside	C16H19N5O6	-4.83	51797213.7
6	389	5.71	Monotropein	C16H22O11	-3.80	46844488.5
7	693	5.81	Methyl 3,4,5-trimethoxycinnamate [arabinosyl-(1->3)-[glucosyl-(1->6)]-glucosyl] ester	C29H42O19	0.18	43832221.9
8	621	8.36	Tolazamide	C14H21N3O3S	2.36	31566436.9
9	641	7.43	Famciclovir	C14H19N5O4	0.87	30737133.3
10	553	8.09	Gibberellin A37 glucosyl ester	C26H36O10	-1.60	30651726.6
11	639	6.42	Didymin	C28H34O14	-0.45	19767532.3
12	623	6.47	Acteoside	C29H36O15	0.09	19487425.6
13	571	6.11	Demethyloleuropein	C24H30O13	1.29	12702192.5
14	282	1.19	8-[(Aminomethyl)sulfanyl]-6-sulfanyloctanoic acid	C9H19NO2S2	-4.38	12212811.0
15	477	7.61	Ala Leu Phe Glu	C23H34N4O7	-4.65	11066566.9
16	725	9.24	Licorice glycoside A	C36H38O16	4.95	10528525.7
17	461	7.87	Neryl rhamnosyl-glucoside	C22H38O10	-1.66	10026632.6
18	533	0.63	3-Fucosyllactose	C18H32O15	1.30	10007452.3
19	745	7.51	Pentacarboxyl porphyrinogen III	C37H40N4O10	3.66	9114090.1
20	493	7.98	Neryl arabinofuranosyl-glucoside	C21H36O10	-1.92	8954593.3
21	393	4.78	Foeniculoside VIII	C16H28O8	-2.70	8892977.2
22	461	8.12	3-O-(alpha-L-rhamnopyranosyl-(1-2)-alpha-L-rhamnopyranosyl)-3-hydroxydecanoic acid	C22H40O11	-1.01	8361152.7
23	739	6.42	Rubrofusarin 6-[glucosyl-(1->3)-glucosyl-(1->6)-glucoside]	C33H42O20	1.62	7937872.9
24	327	8.30	Corchorifatty acid F	C18H32O5	-4.39	7845509.6
25	551	7.06	Caryatin glucoside	C24H26O12	0.79	7582397.4

Rt, retention time; Me, mass error; Rc, relative content.

**Table 2:** Characters of the study participants at baseline and 12 wk.

Characters	Baseline	4 wk	12 wk	P
Weight, kg	70.27±8.75	69.82±8.69	68.50±8.44	<.01*∗*
BMI, kg/m^2^	29.85±3.70	29.67±3.73	29.09±3.54	<.01*∗*
Fat, kg	29.35±6.84	28.64±7.00	27.24±7.12	<.01*∗*
Fat, %	41.33±5.27	40.53±5.74	39.25±6.37	<.01*∗*
Protein, kg	8.04±0.70	8.12±0.69	8.08±0.73	0.46
Muscle, kg	22.23±2.11	22.49±2.11	22.40±2.19	0.41
Viscera fat area, cm^2^	107.65, 32.50	105.20, 37.10	102.40, 28.20	<.01*∗*
Fitness score	67.00, 8.00	67.00, 7.00	68.00, 8.00	0.03*∗*
Abdomen Circumference, cm	95.43±8.19	95.56±8.67	92.26±8.78	<.01*∗*
Hip Circumference, cm	100.88±5.51	100.76±5.47	100.28±5.53	0.45
Metabolic rate, kcal/d	1253.74±76.90	1259.46±76.80	1261.04±82.03	0.59
Physical activity, MET-h/wk	140.88, 162.75	65.13, 70.75	67.90, 0.6.67	0.01*∗*
*Dietary intake per day*				
Energy, kcal	1998.23±529.47	NA	1920.45±611.10	0.62
Protein, g	68.88±20.03	NA	65.67±17.18	0.55
Fat, g	79.56±38.06	NA	69.65±24.57	0.85
Carbohydrate, g	257.36±70.12	NA	261.81±108.78	0.27

Data with normal distribution are presented as mean ± SD and data with nonnormal distribution are presented as median, interquartile range.

P values were calculated by paired T test for normal distribution variables and Wilcoxon signed-rank test for nonparametric tests. We used linear models and Friedman's test to analyze repeated measurement at baseline and 4 and 12 weeks when needed.

*∗*P<0.05.

**Table 3:** Metabolic variables at baseline and 12 wk after *Ligustrum robustum* intervention.

Biomarkers	Baseline	12 wk	P
Lipid profile			
TG, mmol/L	1.57±0.96	1.48±0.87	0.33
CHOL, mmol/L	4.75±0.74	4.72±0.95	0.81
LDL cholesterol, mmol/L	3.00±0.77	3.06±0.80	0.67
HDL cholesterol, mmol/L	1.33±0.27	1.42±0.33	0.01*∗*
Glucose metabolism			
Glucose, mmol/L	5.45, 0.85	5.27, 0.71	<.01*∗*
Insulin, mU/L	9.50, 6.14	9.84, 4.75	0.48
C-Peptide, pmol/mL	0.77, 0.41	0.77, 0.39	0.09
HOMA-IR	2.42, 2.56	2.24, 1.41	0.87
HOMA-*β*, %	87.53, 50.88	103.99, 62.66	<.01*∗*
Cytokines and hormones			
IL6, pg/mL	0.86, 0.47	0.68, 0.79	0.39
IL8, pg/mL	6.04, 4.21	4.79, 2.45	0.02
Leptin, pg/mL	6856.5, 5203.25	5628, 3290.25	<.01*∗*
Safety assessment			
Total protein, g/L	73.67±3.94	73.99±4.36	0.48
Globulin, g /L	28.36±3.27	28.39±3.42	0.91
Albumin/Globulin (A/G)	1.62±0.19	1.63±0.18	0.58
ALB, g/L	45.31±1.79	45.60±1.86	0.30
ALT, U/L	30.90±20.49	22.43±9.76	0.02*∗*
AST, U/L	27.57±11.80	22.03±7.35	0.01*∗*
GGT, U/L	31.37±27.48	25.37±20.77	0.18
DBIL, umol/L	3.70±1.24	3.63±1.09	0.75
IDBIL, umol/L	6.17±2.57	6.50±2.54	0.55
TBIL, umol/L	9.87±3.71	10.13±3.55	0.73
UREA, mmol/L	5.30±1.33	5.44±1.37	0.59

Data with normal distribution are presented as mean ± SD and data with nonnormal distribution are presented as median, interquartile range.

P values were calculated by paired T test for normal distribution variables and Wilcoxon signed-rank test for nonparametric tests.

ALB, albumin; ALT, alanine aminotransferase; AST, aspartic transaminase; CHOL, total cholesterol; DBIL, direct bilirubin; GGT, gamma-glutamyl transferase; HDL, high-density lipoprotein cholesterol; LDL, low-density lipoprotein cholesterol; TBIL, total bilirubin; TG, triglycerides; IDBIL, Indirect bilirubin.

*∗*P<0.05.

## Data Availability

The data used to support the findings of this study are available from the corresponding author upon request.
